# Effect of Microalloying Rare-Earth Nd on Microstructure Evolution and Mechanical Property of Cu Alloy

**DOI:** 10.3390/ma17205112

**Published:** 2024-10-19

**Authors:** Mingyi Zhang, Jichun Yang, Chongyuan Huang, Puyou Ying, Yong Huan, Fei Liu

**Affiliations:** 1 School of Rare Earth Industry, Inner Mongolia University of Science and Technology, Baotou 014010, China; zmyymjr@126.com (M.Z.); 15384850013@163.com (J.Y.); 2 Key Laboratory of Green Extraction & Efficient Utilization of Light Rare-Earth Resources, Inner Mongolia University of Science and Technology, Ministry of Education, Baotou 014010, China; 3 Inner Mongolia Institute of Metal Materials, Baotou 014034, China; 4 State Key Laboratory of Nonlinear Mechanics (LNM), Institute of Mechanics, Chinese Academy of Sciences, Beijing 100190, China; huangchongyuan24@mails.ucas.ac.cn; 5 School of Engineering Science, University of Chinese Academy of Sciences, Beijing 100049, China; 6 International Joint Institute of Advanced Coating Technology, Taizhou University, Taizhou 318000, China; ypu@tzc.edu.cn

**Keywords:** Cu alloy, mechanical property, microalloying, Nd, strength–ductility trade-off

## Abstract

Cu alloys have been widely used in the manufacture of liners because of their high density, good plasticity, and excellent thermal conductivity. In order to achieve excellent jet stability and penetration performance, it is necessary to further improve the mechanical properties of Cu-based liners. Nevertheless, the simultaneous enhancement of strength and ductility of the Cu alloys remains a huge challenge due to the strength–ductility trade-off phenomenon of metals/alloys. In this study, the microstructure evolution of rare earth Nd-modified Cu alloy and its effect on mechanical properties were investigated using OM, SEM, EBSD, and TEM techniques. The results show that the ultimate tensile strength (218 MPa) and elongation (50.7%) of sample 1 without Nd are the lowest. With increasing Nd content; the tensile strength and elongation of the samples increase; and the mechanical properties of sample 4 are the best, with a tensile strength of 278.6 MPa and elongation of 65.2%. In addition, with the increase in Nd content, not only is the grain size of the Cu-Nd alloy refined, but also the strength and plasticity are improved so that the strength–ductility trade-off phenomenon is improved. The strength improvement is mainly attributed to grain refinement strengthening, dispersion strengthening, and strain hardening. The increase in ductility is mainly related to the improvement of the microstructure heterogeneity by the Nd element.

## 1. Introduction

Due to its high density, good plasticity, fast sound velocity, and excellent thermal conductivity, Cu alloys have been widely used in the manufacture of liners, in aerospace, in the heat sink of the diverter in ITER, and in future nuclear reactors [1,2,3,4]. However, with the continuous development of modern industrial technology, more elevated demands have been imposed on the mechanical properties of copper-based liners. In order to improve the jet stability and penetration performance of Cu-based liners, it is necessary to further improve the mechanical properties of the Cu matrix. Therefore, it is urgent to study the microstructure evolution of the Cu alloy and its influence on mechanical properties in order to improve the jet stability and penetration performance of the alloy.

Generally, it is difficult for metallic materials to simultaneously obtain high-strength and excellent ductility (strength–ductility trade-off), which brings great challenges to the development and preparation of high-strength and high-toughness metal materials [5,6,7]. Studies have shown that grain refinement strengthening is a method that can improve both strength and plasticity [8,9,10]. There are a lot of studies on the use of heat treatment or post-treatment technology to refine the grain size of Cu alloys [11,12,13]. Li et al. refined the grain size of Cu-based liners by vacuum gradient heat treatment [11]. The results show that refining grain size and reducing texture intensity can improve the mechanical properties of the Cu-based coating [11]. Elshenawy et al. studied the effect of current intensity on the mechanical properties of Cu-based liner and found that the current intensity significantly affects the grain size of the Cu matrix, and the penetration depth of the liner after optimizing the grain size is increased by 22.7% [12]. Yu et al. studied the effect of shaped charge explosion on the grain refinement of Cu alloy, and proposed the homogenous nucleation (i.e., rotational-mode) mechanism of dynamic recrystallization to explain the microcrystal transformation process during shaped charge [13]. In addition to refining the grain size of Cu alloy through heat treatment and post-treatment technology, alloying of rare earth elements is another effective method to refine the grain size [14,15]. Zhang et al. studied the effect of the Nd element on the grain size and mechanical properties of a Cu-Al-Ni alloy [14]. The results show that after adding the Nd element, the grain size of the Cu alloy is refined from millimeters to microns, and the refinement of the grain size effectively improves the mechanical properties of the Cu alloy [14]. Cui et al. studied the effects of the rare earth elements La, Ce, and Nd on the microstructure evolution and mechanical properties of a Cu-Sn-Ti alloy, and found that the addition of rare earth elements improved the microstructure of the Cu alloy; reduced the number of cracks, holes, and other defects in the alloy; and also reduced the size of these defects. Thus, the mechanical properties of the Cu alloy are improved [15]. It is worth noting that compared with the other two rare earth-modified Cu alloys, the mechanical properties of the Cu-Nd alloy have improved the most significantly [15]. It can be seen that adding rare-earth Nd elements to Cu alloys can not only refine the grain size but also reduce the number of defects so as to achieve a great improvement in the mechanical properties.

Aiming at the great demand for improving the mechanical properties by adjusting the microstructure of Cu-based liners, in this study, the microstructure evolution and mechanical properties of the Cu alloy with different Nd content were investigated using optical microscopy (OM), scanning electron microscopy (SEM), the electron back-scatter diffraction (EBSD) technique, and transmission electron microscopy (TEM). The improvement mechanisms of strength–ductility trade-off and effect mechanisms of Nd content on the evolution of microstructure and texture were revealed. 

## 2. Materials and Methods

The chemical composition of the Cu alloy in this study is given in Table 1. An Nd-Cu ingot was melted in an induction furnace (ZGJL0.030-100-2.5B2, supplied by Jinzhou Jinkai Electric Furnace Co., Ltd., Jinzhou, China) at 1220 °C. Because the Nd-Cu alloy is not easy to oxidize and the melting point is close to Cu, Nd was added in the form of Nd-Cu intermediate alloy. The content of the Nd element in prepared Cu-Nd alloy was measured by inductively coupled plasma mass spectrometer (7800 ICP-MS, supplied by Agilent, Beijing, China). Four kinds of Cu-Nd alloys were prepared, these Cu-Nd alloys were named as sample 1, sample 2, sample 3, and sample 4.

**Figure 1:**
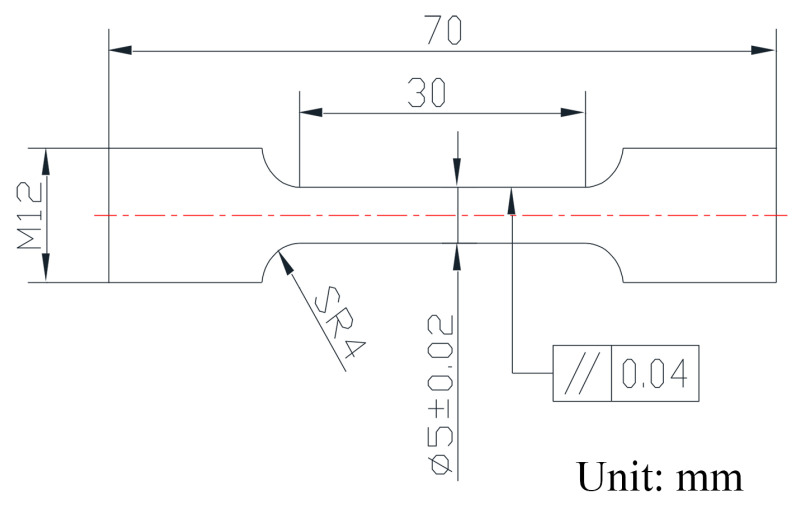
Tensile sample configuration and size.

The metallographic microstructure was observed by an optical microscope (OM) with the model Axio Observer A1 Zeiss, supplied by Carl Zeiss AG, Oberkochen, Germany. The samples were etched in FeCl_3_-HCl (30%) solution, which was eroded for 15~20 min. SEM with the models TESCAN MIRA LMS (supplied by TESCAN, Brno, Czech Republic) and ThermoFisher Apreo were equipped with an EDS (energy dispersive spectrometer, supplied by Thermo Fisher Scientific, Waltham, MA, USA) to observe the fracture morphology and analyze the chemical composition. FEI Sirion 400NC SEM (supplied by FEI Company, Hillsboro, OR, USA) was used to collect EBSD data. The microstructure and crystal structure were characterized by Tecnai G2 F30 S-TWIN TEM (supplied by FEI Company, Hillsboro, OR, USA). The TEM accelerated voltage was 300 kV. TEM samples were prepared by a twin-jet polishing instrument with the model TJ100-SE-TMS (supplied by Jiangsu Leibo Scientific Instrument Co., Ltd., Wuxi, China). The electrolyte was a nitrate methanol solution with a volume ratio of 1:3. The temperature was −30 °C, the voltage was 12–13 V, and the electrolytic current was 26 mA. The CMT5305 electronic universal testing machine (supplied by MTS Systems Corporation in Shanghai, China) was used to test the mechanical properties of the alloy. The tensile samples were rod-like samples, the size of which is shown in Figure 1.

## 3. Results

### 3.1. Mechanical Property

**Figure 2:**
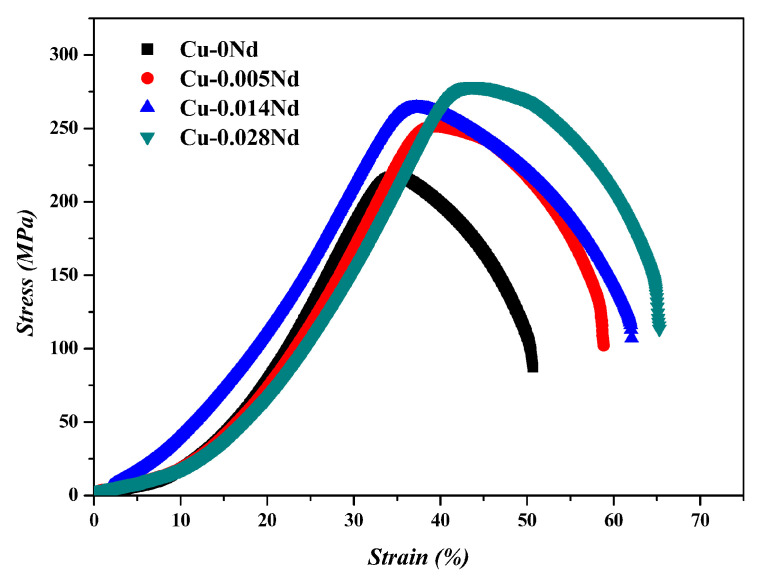
Tensile properties of Cu alloys with different Nd contents at room temperature.

Figure 2 shows the tensile properties of the Cu-Nd alloys with different Nd at room temperature. As shown in Figure 2, the ultimate tensile strength (218 MPa) and elongation (50.7%) of sample 1 without the Nd element are the lowest. With the increase in Nd, the tensile strength and elongation increase, and the mechanical properties of sample 4 are the best, with a tensile strength of 278.6 MPa and elongation of 65.2%. The addition of the Nd element greatly improves the mechanical properties, which indicates that adding the Nd element to Cu alloy can effectively improve the strength–ductility trade-off phenomenon.

### 3.2. Optical Microstructure

**Figure 3:**
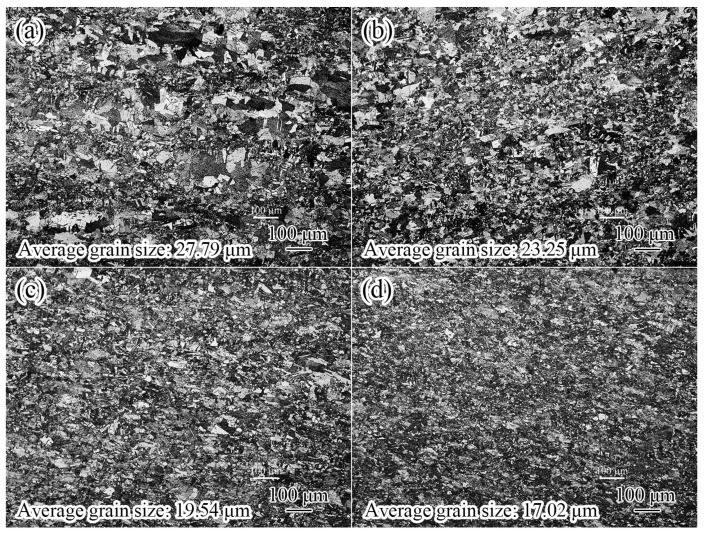
Optical images of Cu alloys with different Nd contents: (**a**) sample 1; (**b**) sample 2; (**c**) sample 3; (**d**) sample 4.

The optical microstructures of the samples with different Nd are shown in Figure 3. It can be seen from Figure 3 that the optical microstructures of different samples are composed of equiaxed grains and twins. The grain size of sample 1 is very inhomogeneous, both large polygonal grains are formed, and fine equiaxed grains are observed. With the increasing Nd content, the grain shape, size, and distribution gradually become uniform, and the grain size is significantly refined from 27.79 μm to 17.02 μm, which indicates that adding Nd element can not only refine the grain size but also improve the uniformity of the grain structure. 

### 3.3. SEM Characterization

**Figure 4:**
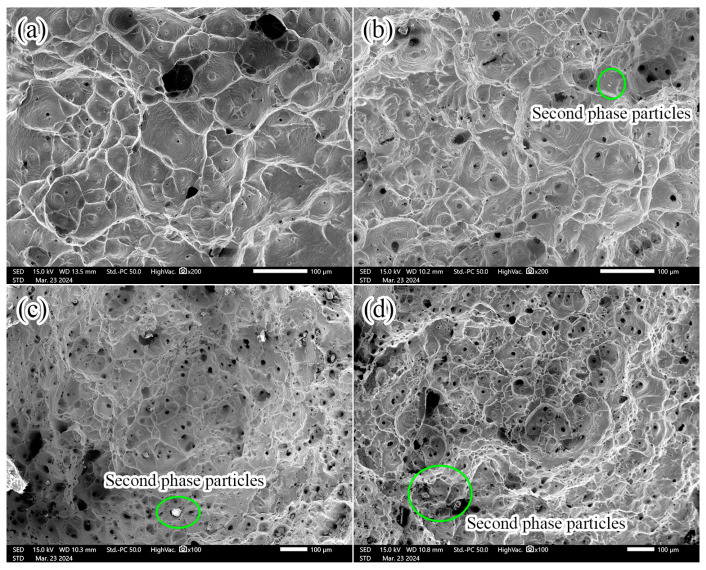
Fracture morphology of Cu alloys with different Nd contents: (**a**) sample 1; (**b**) sample 2; (**c**) sample 3; (**d**) sample 4.

**Figure 5:**
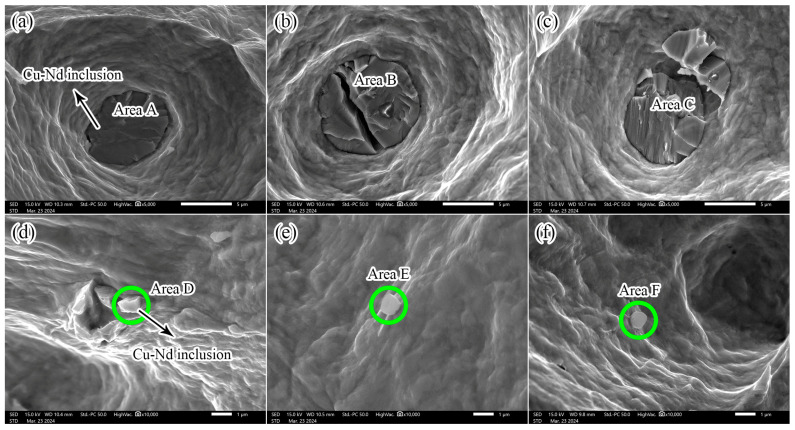
Fracture behavior of Cu-Nd inclusion in Cu alloys with different Nd contents: (**a**,**d**) sample 2; (**b**,**e**) sample 3; (**c**,**f**) sample 4.

Figure 4 shows the tensile fracture morphology of the Cu alloys with different Nd. It can be seen from Figure 4 that a large number of dimples are formed in the fractures of different samples, indicating that the fracture mechanism of the Cu-Nd alloy is a ductile fracture. In addition, with the increasing Nd, the size and number of the dimples gradually homogenize, which improves the ductility of the Cu-Nd alloy. Compared with sample 1 without Nd, the broken micron scale second phase particles were also observed in samples 2–4, as shown in Figure 4b–d. In order to analyze the fracture behavior and chemical composition of these second-phase particles, SEM images, and EDS results of their fracture surfaces were collected, as shown in Figure 5. There are two scales of the second phase particles, one is large, about 5 μm, and the other is relatively small, less than 1 μm. It is worth noting that the larger particles are located at the bottom of the dimple and have cleavage fracture characteristics, while the smaller particles are located at the edge of the dimple and have intergranular fracture characteristics. The fracture mechanism of both particles is a brittle fracture. The results of Figure 5a–c show that brittle fracture of the coarse second-phase particles during tensile deformation is the main reason for the failure of the Cu-Nd alloy.

The EDS results of the second phase particles in Figure 5 are given in Table 2. As shown in Table 2, the particles of both sizes are Cu-Nd inclusions. Smaller particles have higher C and O content than larger particles. The different C and O in the second phase particles of the two sizes may lead to different brittle fracture mechanisms, that is, the particles with large sizes show cleavage fracture characteristics and the particles with small sizes show intergranular fracture characteristics.

### 3.4. EBSD Analysis 

The elastic stiffness, Schmid factor, Taylor factor, and KAM value of the Cu-Nd alloys were calculated, as shown in Table 3. The elastic stiffness and Schmid factor of the alloys do not change significantly, indicating that the addition of the Nd element little affects the slip behavior of the dislocation. With the increasing Nd, the Taylor factor first decreases, then increases, and finally decreases. The change in the Taylor factor is mainly related to the texture evolution. It is worth noting that the Nd content significantly affects the KAM value of the alloy. When a trace Nd element is added, the KAM value of the Cu matrix can be significantly reduced, and with the increase in Nd content, the KAM value of the matrix increases gradually to 0.40 at first and then sharply to 1.97. The KAM value directly reflects the degree of strain concentration in the matrix, and the greater the KAM value, the greater the degree of strain concentration.

**Figure 6:**
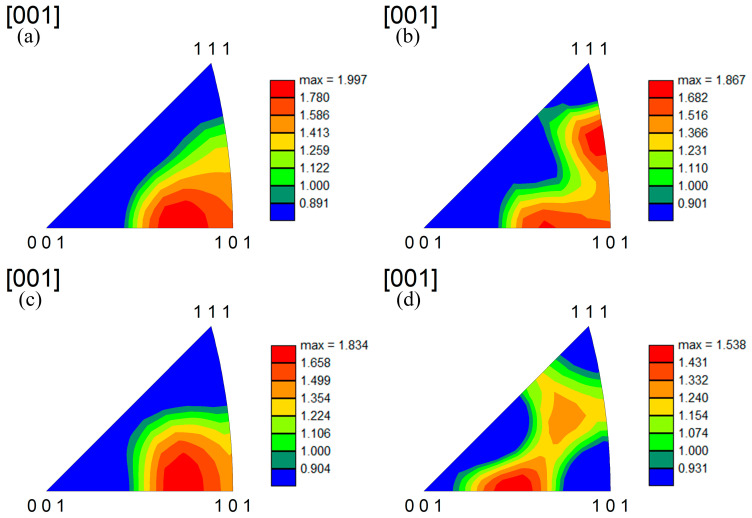
Inverse pole figure of Cu alloys with different Nd contents: (**a**) sample 1; (**b**) sample 2; (**c**) sample 3; (**d**) sample 4.

Figure 6 is a normal direction (ND) IPF figure of the Cu alloys. As shown in Figure 6, the ND//[101] texture is formed in sample 1, and after adding the trace Nd element, approximately ND//[111] texture and ND//[001] textures are formed in the alloy (Figure 6b). However, with the increase in Nd content, the ND//[101] texture is formed. The ND//[111] texture and the ND//[001] texture evolved into the ND//[101] texture (Figure 6c). With the further increase in Nd content, the grain orientation gradually tends to randomization (Figure 6d). By comparing Table 3 and Figure 6, the evolution trend of the Taylor factor of the alloy is correlated with the evolution law of the texture. Our previous studies have shown that the grains with different orientations have different Taylor factors, which leads to different deformation resistance of the grains with different orientations [16,17]. Therefore, the alloy matrix will be hardened or softened, that is, the alloy strength will be increased through texture strengthening, or the strength will be reduced through texture softening.

### 3.5. TEM Observation

**Figure 7:**
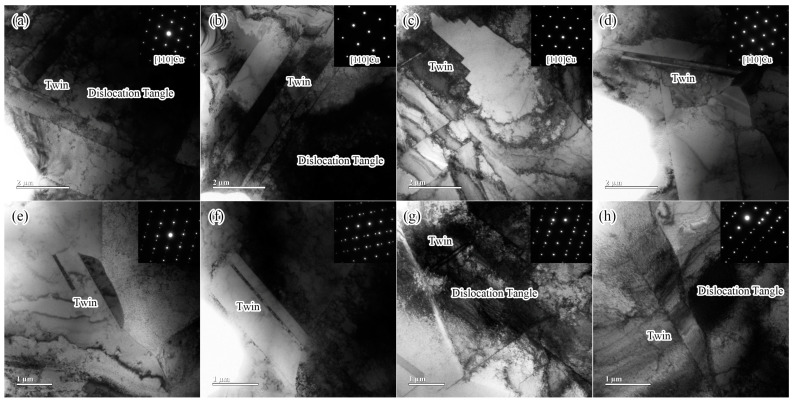
TEM images of Cu alloys with different Nd contents: (**a**) TEM microstructure of sample 1; (**b**) TEM microstructure of sample 2; (**c**) TEM microstructure of sample 3; (**d**) TEM microstructure of sample 4; (**e**) twin structure of sample 1; (**f**) twin structure of sample 2; (**g**) twin structure of sample 3; (**h**) twin structure of sample 4.

TEM images of the Cu alloys are shown in Figure 7. As shown in Figure 7, a large number of twins and dislocations were observed in the four Cu-Nd alloys. With the increasing Nd content, the number of twins increases gradually, the interaction between the dislocation and the twin is enhanced, and a large number of dislocation tangles are formed near the twin. In addition, the increase in Nd content leads to the morphology evolution of the twins from sheetlike twins to large-sized step-like twins, as shown in Figure 7c,d. It is worth noting that according to the selected area electron diffraction (SAED) pattern of <110> direction, no second-phase particles are formed. Studies have shown that rare earth elements mainly appear in the form of large-sized inclusions in Cu alloys [18,19], so no small-sized second-phase particles are observed at the TEM scale.

## 4. Discussion

### 4.1. Effect of Nd Element on Microstructure Evolution of Cu Alloy

The influence of the Nd element on the microstructure of the Cu alloy mainly includes the following aspects. (1) Grain refinement. After the addition of the Nd element, Cu-Nd inclusions are formed (Figure 3, Figure 4 and Figure 5), which can effectively inhibit grain boundary migration and further refine the grain size. In addition, with the increasing Nd, the number of Cu-Nd inclusions increased, further refining the grain size. (2) The Nd element significantly affects texture evolution. As shown in Figure 6, an approximate ND//[101] texture is formed in sample 1 without the Nd element. Besides the ND//[101] texture formed in sample 2 after the addition of trace Nd element, the [111] direction of some grains gradually turns to the normal direction. With further increases in Nd content, the alloy texture first evolves into the ND//[101] texture and then gradually tends to randomization. In addition, it can be seen from Table 3 that different types of textures have different Taylor factors, which indicates that different types of textures make different contributions to strength. Since samples 1 and 3 both form an approximate ND//[101] texture, the two samples have approximately equal Taylor factors: the Taylor factor of sample 1 is 3.08, and that of sample 3 is 3.07. The slight difference in the Taylor factor is related to the texture intensity. Since sample 2 forms a texture of other orientations in addition to the approximate ND//[101] texture (Figure 6b), this results in a decrease in the number of grains with ND//[101] orientations, leading to a smaller Taylor factor (3.04). In addition, because the texture of sample 4 tends to be randomized, its Taylor factor is the smallest, which is 3.03. In addition, the larger the Taylor factor of the grain, the more significant the strengthening effect [16,17]. Thus, compared with other orientations, the ND//[101] texture can improve the alloy strength by texture strengthening. (3) The Nd element can improve the microstructure heterogeneity and plasticity of the Cu-Nd alloy. As shown in Figure 3, sample 1 without the Nd element has a mixed grain structure, showing strong microstructure heterogeneity. With the increasing Nd, the grain size is gradually refined and tends to be uniform. Because the Nd element improves the heterogeneity of the microstructure, the strain concentration in the alloy matrix is significantly reduced, as shown in Table 3 (KAM). It is worth noting that when the content of the Nd element reaches 0.028%, the strain concentration of the alloy matrix increases significantly, and the increase in strain concentration is mainly related to the formation of the Cu-Nd inclusions. In addition, the ductility of the Cu-Nd alloy increases with the increase in Nd content. (4) Promotes the formation of large-sized step-like twins. Studies have shown that the Nd element can not only reduce the stacking fault energy of close-packed hexagonal alloy but also reduce the stacking fault energy of face-centered cubic alloy [20,21]. Due to the decrease in the stacking fault energy, the width of the extended dislocation increases, thus inhibiting the cross-slip and climb of the dislocation. In order to release local stress concentration, the alloy will coordinate deformation through the twining mechanism [22].

### 4.2. Strengthening Mechanism of Nd-Modified Cu Alloy

When the grain size is greater than 1 μm, the contribution of grain refinement strengthening to strength can be described by the Hall–Petch relationship [23,24,25]:

(1) $$\sigma_{GB}=kd^{-\frac{1}{2}}$$

where k is constant (k = 0.14 $\mathrm{MPa}\;m^{\frac{1}{2}}$) [23], and d is the average grain diameter. The contribution of grain refinement strengthening is given in Table 4. These results are consistent with Figure 2 and Figure 3. 

Although the Cu-Nd inclusions tend to crack in the deformation process, thus deteriorating the plasticity of the alloy, the Cu-Nd inclusions can also impede grain boundary migration to refine the grain size of the alloy. In addition, as shown in Figure 4, Figure 5 and Figure 6, the shape of the Cu-Nd inclusions is mostly spherical or granular, so that the Cu-Nd inclusions can interact with dislocations during tensile deformation, thereby strengthening the alloy. The contribution of the Cu-Nd inclusion to strength can be approximated by the Orowan stress criterion [26]:

(2) $$\tau_{Rowan}=\frac{0.4MGb}{\pi \lambda }\frac{\ln\left(\frac{2\bar{r}}{b}\right)}{\left(\sqrt{1-\nu }\right)}$$

where $\tau_{Rowan}$ is the Orowan stress. M is the Taylor factor (M=3.06 [26]). G  is the shear modulus (G=45  [23]). b is Burgers vector (b=0.256 nm [23]). $\bar{r}$ is the mean radius of a circular section in a random plane for a sphere second phase particle and $\bar{r}=\sqrt{\frac{2}{3}}r$. r is the average radius of the second phase particle, and λ is the edge-to-edge inter-precipitate spacing. λ can be expressed as [26]:

(3) $$\lambda =2\bar{r}\left(\sqrt{\frac{\pi }{4f}}-1\right)$$

where f is the volume fraction of second-phase particles. 

As shown in Table 3, the strain of the alloys is different, and, therefore, strain hardening is also a strengthening mechanism in this study. Strain hardening is mainly a strengthening effect caused by the interaction between dislocations. The interaction between dislocations can be calculated using the Bailey–Hirsch relationship [27,28]:

(4) $$\tau_{Dis}=M\alpha Gb\sqrt{\rho_{d}}\;$$

where M is the Taylor factor. α is constant (α=0.2 [29]). $\rho_{d}$ is the dislocation density. 

## 5. Conclusions and Future Research Directions

In this study, the effect of rare earth Nd content on the microstructure evolution and its mechanical properties of the Cu-Nd alloy were investigated using OM, SEM, EBSD, and TEM techniques, and the following conclusions were obtained:

(1) With the increase in rare earth Nd content, the ultimate tensile strength and elongation are increased, and the strength–ductility trade-off phenomenon is improved. The increased strength is attributed to grain refinement strengthening, dispersion strengthening, and strain hardening, and the excellent ductility is mainly related to the improvement of the microstructure heterogeneity by the Nd element.

(2) With the increasing Nd, not only is the grain size refined, but also the formation of twins is promoted. The main reason for grain refinement is that the Cu-Nd inclusions can effectively inhibit grain boundary migration after adding the Nd element. The formation of the twin is mainly related to the fact that the stacking fault energy is decreased by adding the Nd element, inhibiting the cross-slip of dislocation.

(3) The ND//[101] texture is formed in the sample without the Nd element, and the approximately ND//[111] and ND//[001] textures are formed after the addition of the trace Nd element, but with the increase in Nd, the ND//[111] and ND//[001] textures evolve into the ND//[101] texture. With the further increase Nd, the texture tends to randomization.

The influence of the Nd element on the physical properties (e.g., electrical conductivity, thermal conductivity) of copper is worthy of further study.

## Figures and Tables

**Table 1:** Chemical composition of experimental materials (mass fraction, wt.%).

Sample/Element	Se	Te	Bi	P	S	Sb	O	As	Nd	Cu
Sample 1	0.0002	0.0002	0.0002	0.0001	0.0003	0.0004	0.0008	0.0005	--	Remainder
Sample 2	0.0002	0.0002	0.0002	0.0001	0.0003	0.0004	0.0008	0.0005	0.005	Remainder
Sample 3	0.0002	0.0002	0.0002	0.0001	0.0003	0.0004	0.0008	0.0005	0.014	Remainder
Sample 4	0.0002	0.0002	0.0002	0.0001	0.0003	0.0004	0.0008	0.0005	0.028	Remainder

**Table 2:** EDS results of the fractured second-phase particles in Figure 5.

Area/Element (at.%)	C	O	Nd	Cu
A	5.65	3.20	12.69	78.46
B	5.87	1.12	0.40	92.61
C	6.95	5.09	11.43	76.53
D	33.58	11.63	0.18	54.61
E	23.59	10.21	11.55	54.65
F	12.01	26.43	23.95	37.61

**Table 3:** EBSD calculating results.

Sample	Elastic Stiffness	Schmid Factor	Taylor Factor	KAM
Sample 1	112.24	0.45	3.08	0.75
Sample 2	110.49	0.45	3.04	0.22
Sample 3	106.79	0.45	3.07	0.40
Sample 4	111.23	0.45	3.03	1.97

**Table 4:** Contribution of grain refinement to strength.

Alloy Number	Grain Size (μm)	$d^{-\frac{1}{2}}$ $\left(m^{-\frac{1}{2}}\right)$	$\sigma_{GB}$ (MPa)
1	27.79	189.69	26.56
2	23.25	207.39	29.03
3	19.54	226.22	31.67
4	17.02	242.39	33.93

## Data Availability

The original contributions presented in the study are included in the article.
